# Pain relief associated with decreased oxyhemoglobin level in left dorsolateral prefrontal cortex

**DOI:** 10.1371/journal.pone.0256626

**Published:** 2021-08-23

**Authors:** Shun Miyashiro, Yurika Yamada, Masaru Nagaoka, Rei Shima, Toshizumi Muta, Haruyuki Ishikawa, Tetsuri Abe, Masashi Hori, Kotaro Oka, Fusako Koshikawa, Etsuro Ito

**Affiliations:** 1 Department of Biology, Waseda University, Tokyo, Japan; 2 Department of Psychology, Waseda University, Tokyo, Japan; 3 Department of Culture, Media and Society, Waseda University, Tokyo, Japan; 4 Department of Educational Psychology, Waseda University, Tokyo, Japan; 5 Department of Bioscience and Informatics, Keio University, Yokohama, Japan; 6 Waseda Research Institute for Science and Engineering, Waseda University, Tokyo, Japan; 7 Graduate Institute of Medicine, Kaohsiung Medical University, Kaohsiung, Taiwan; Tokai University, JAPAN

## Abstract

Pain in the elbow, shoulder, knee, lower back, and various other joints is relieved by adhesion of pyramidal thorn patches. To elucidate the pain relief mechanism induced by the patches, we established a quantitative method for estimating the pain reduction and investigated the brain regions that change in association with pain relief. We first attempted to quantify the pain relief using transcutaneous electric stimulation (TCES) and a visual analog scale (VAS), and then applied near-infrared spectroscopy (NIRS) to the prefrontal cortex, including the dorsolateral prefrontal cortex (DLPFC) and the orbitofrontal cortex (OFC). We also examined the salivary oxytocin levels, which are thought to reflect oxytocin secretion levels from the posterior pituitary in the brain. Application of pyramidal thorn patches to pain regions decreased the pain degree estimated using TCES and VAS. Oxyhemoglobin levels were likely to be decreased in the left DLPFC on the basis of NIRS measurements during patch treatment, suggesting that the left DLPFC is involved in pain relief. On the other hand, the salivary oxytocin levels varied widely. A potential reason for the varying salivary oxytocin levels is its utilization in the pain region as an analgesic agent. Our results suggest that the left DLPFC will become a target brain region for pain therapy.

## Introduction

Pain relief is highly sought after, especially by those with chronic pain. Recognition of the limitations of commercially available analgesic drugs has led to an increased use of complementary and integrative health approaches (*e*.*g*., natural products, deep breathing, meditation, massage, *etc*.) for various types of pain, such as back pain, neck pain, and joint pain [1]. One integrative approach is therapeutic touch. Saito et al. recently developed a circular adhesive patch with a silicone spine shaped like a pyramidal thorn [2, 3], called a pyramidal thorn patch. Application of pyramidal thorn patches to various pain regions in 300 patients relieved pain in almost all the patients within 4 treatments [2]. In these studies, the degree of pain relief was estimated using a visual analog scale (VAS).

The prefrontal cortex is strongly implicated as a brain region involved in the modulation of both nociception and antinociception [4]. The prefrontal cortex has connections with the insular cortex and medial thalamic nuclei, which are involved in nociception modulation [5–7]. The medial prefrontal cortex receives ascending nociceptive input, but also exerts important top-down control of pain sensation [8]. The medial prefrontal cortex has sustained tonic facilitatory action (*i*.*e*., a noxious thermally-evoked response) over the thalamic centralis lateralis nucleus [9]. The prefrontal cortex is also thought to function in antinociception through connections with the amygdala and periaqueductal grey matter [10–12]. For example, the pathways from the basolateral nucleus of amygdala to the prefrontal cortex inhibit nociceptive cell activities in the prefrontal cortex [13]. We thus hypothesized that observing changes in prefrontal cortex activation offers the opportunity to clarify pain relief mechanisms in the brain.

Near-infrared spectroscopy (NIRS) is a useful, noninvasive method for measuring changes in brain activity [14]. NIRS measures oxyhemoglobin and deoxyhemoglobin concentrations at the brain surface in real-time [15]. NIRS overcomes many problems of other brain imaging techniques, such as functional magnetic resonance imaging (fMRI) or positron emission tomography (PET), including concerns about participant posture, spatial resolution, time resolution, loud sounds, and the need for a shielded room [16]. When NIRS is applied to the prefrontal cortex, activity changes are easily observed as a change in oxyhemoglobin concentrations [15]. Thus, NIRS is useful for exploring brain regions in the prefrontal cortex involved in pain relief.

The effect of oxytocin for pain relief should also be considered [17–19]. Oxytocin is a peptide hormone comprising 9 amino acids, and is synthesized in neurons of the supraoptic nucleus and paraventricular nucleus of the hypothalamus after specific stimulation of the brain [20]. These neurons project to the posterior pituitary, where oxytocin is released into the blood for delivery to peripheral tissues as well as into the brain [21, 22]. Oxytocin has antinociceptive effects [23, 24], but the molecular and cellular mechanisms of pain relief by oxytocin have not been established. We hypothesized that oxytocin secretion is increased during pain relief [19].

In the present study, we first attempted to establish a quantitative method for measuring pain reduction by application of pyramidal thorn patches using transcutaneous electric stimulation (TCES). Changes in oxyhemoglobin levels before and after pain relief in the prefrontal cortex were then examined by NIRS. Finally, salivary oxytocin levels were measured during pain relief by enzyme-linked immunosorbent assay (ELISA). The present study provides insight into both the use of NIRS for quantifying pain relief and the role of oxytocin in pain relief.

## Materials and methods

### Ethics statement

The present study was carried out in accordance with the recommendations of the principles and guidelines of the Declaration of Helsinki. All participants provided written informed consent to participate in the present study. A questionnaire was distributed to the participants to inquire about the pain regions in the body and the time elapsed from the onset of pain. The protocol was approved by the academic research ethics review committee of Waseda University, Office of Research Ethics (2016–282, 2018–067, 2021–018).

### Participants

Two groups of participants were enrolled. The first group comprised 18 voluntary participants from the Tokyo area in Japan who did not have pain on the basis of self-assessment (no-Pain group; age 18‒29 years; 10 men and 8 women; all right-handed). The second group comprised 27 voluntary participants who declared themselves to have pain on the basis of self-assessment (Pain group; age 19‒25 years; 10 men and 17 women; all right-handed). The pain regions in these participants were as follows: shoulder joint (n = 13); lower back (n = 11); knee joint (n = 1); sternum (n = 1); and left wrist (n = 1). Each participant in the Pain group stated the type of posture that produced the pain.

### Treatment with the pyramidal thorn patch

**Fig 1:**
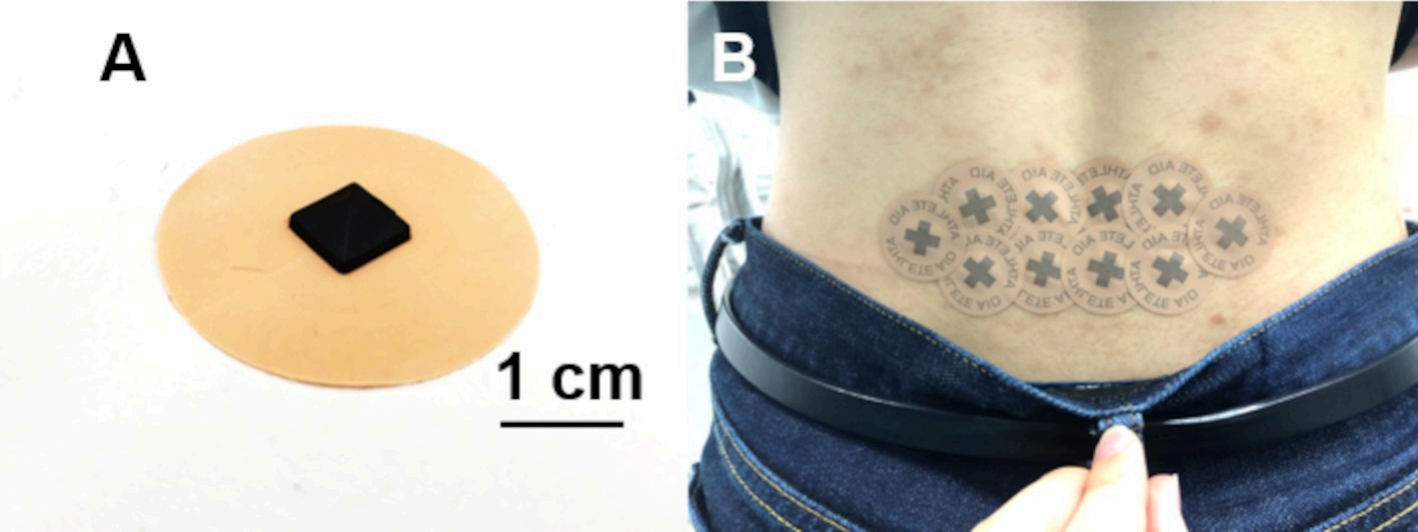
Pyramidal thorn patch. ***A*.** The pyramidal thorn patch has a diameter of 3 cm and a thickness of 0.1 mm, and the tapered pyramidal thorn made of low-density polyethylene is 3 mm high and a square with 7-mm sides. ***B*.** Application of pyramidal thorn patches to the pain region (*e*.*g*., lower back). After palpating to determine the pain region, several pyramidal thorn patches were applied to alleviate pain.

The pyramidal thorn patch was developed by Saito and colleagues [2, 3]. The detailed method for the patch treatment was described previously [3]. Briefly, the pyramidal thorn patch is a circular adhesive patch (3 cm in diameter and 0.1-mm thick) made of synthetic resin (Fig 1). The center of the circular patch is made of low-density polyethylene in a 7-mm square area and the tapered pyramidal thorn is 3 mm high. To determine where to place the pyramidal thorn patches, the experimenters used strong thumb pressure to palpate the skin surface of the pain region and its surrounding area indicated by the participant. After the experimenters determined the pain location, several pyramidal thorn patches were adhered in the region. For the Pain group, 15–20 pyramidal thorn patches were adhered to the pain regions; and for the no-Pain group, approximately 15 patches were adhered over the shoulder joint. The duration of patch treatment was about 30 min.

### Pain measurements by TCES and VAS

**Fig 2:**
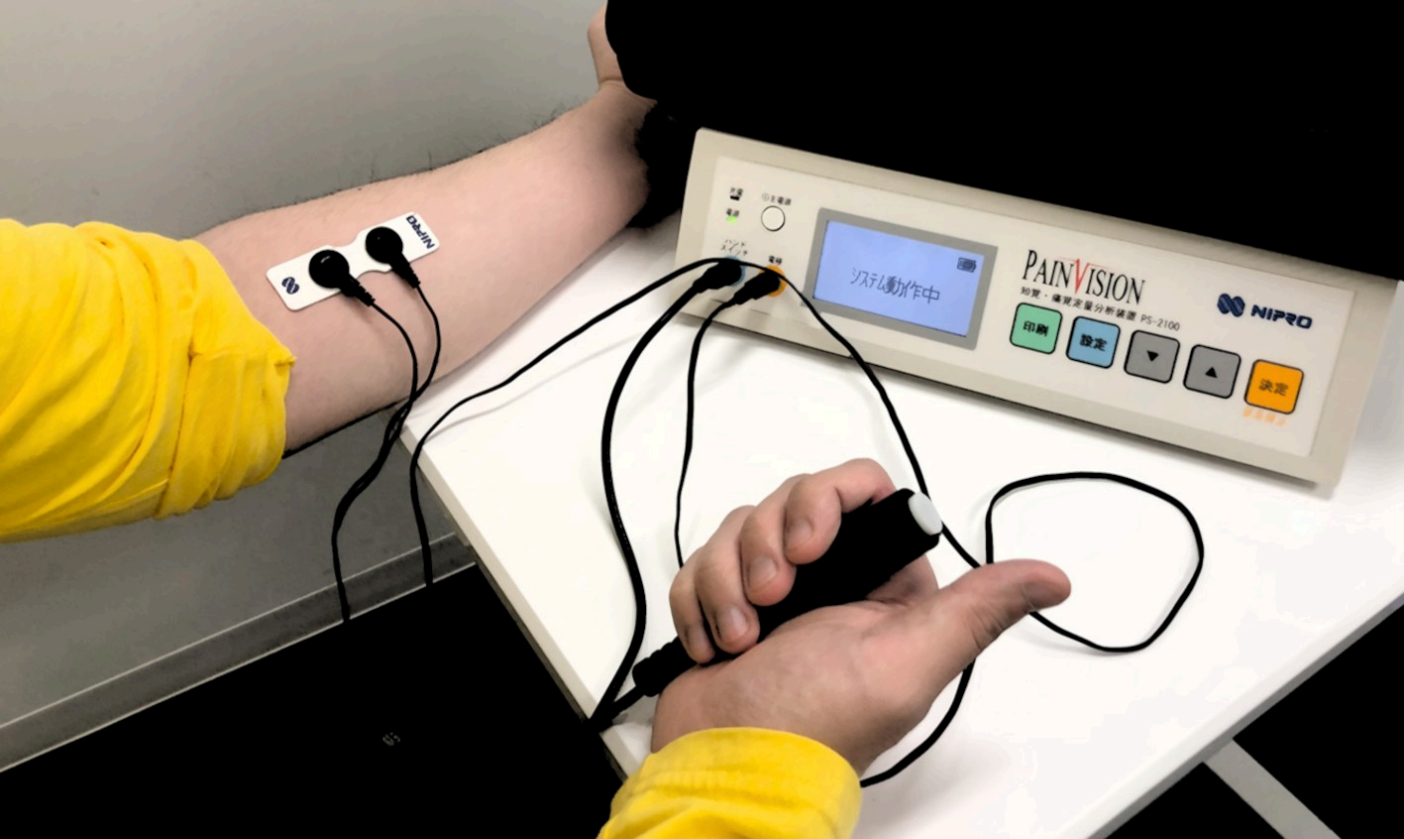
Transcutaneous electric stimulation (TCES). The TCES shows the ‘pain degree’, calculated by: [(pain-equivalent current–minimum perceived current) / minimum perceived current] × 100.

Before and after patch treatment, the degree of pain was determined using a TCES and VAS. The directions of posture that produced the pain were given to the participants in the Pain group with an audio guide. The TCES (Pain Vision, PS 2100, NIPRO, Osaka, Japan) shows the ‘pain degree’ that can be calculated from the current perception threshold, defined as the lowest electrical current detected by participants, and the pain-compatible electrical current, defined by the electrical current judged by each participant as being compatible with the intensity of ongoing pain (Fig 2) [25]. The 10-s TCES measurements were performed 3 times each before and after the patch treatment, and the average of the 3 measurements was calculated. The pain degree was calculated using the following formula: [(pain-equivalent current–minimum perceived current) / minimum perceived current] × 100. For the no-Pain group, the differences between the minimum pain-evoking current and the minimum perceived current were measured before and after the patch treatment. The VAS (integer scale: 0–100) is a common pain scale for ‘so-called quantification of pain’ [26]. The VAS measurements were performed 3 times each before and after the patch treatment, and the average of the 3 measurements was calculated. We did not apply the VAS measurements to the no-Pain group because they were not experiencing pain before the patch treatment. The period between the onset of patch adhesion and the TCES and VAS measurements was about 5 min.

### NIRS oxyhemoglobin measurements

**Fig 3:**
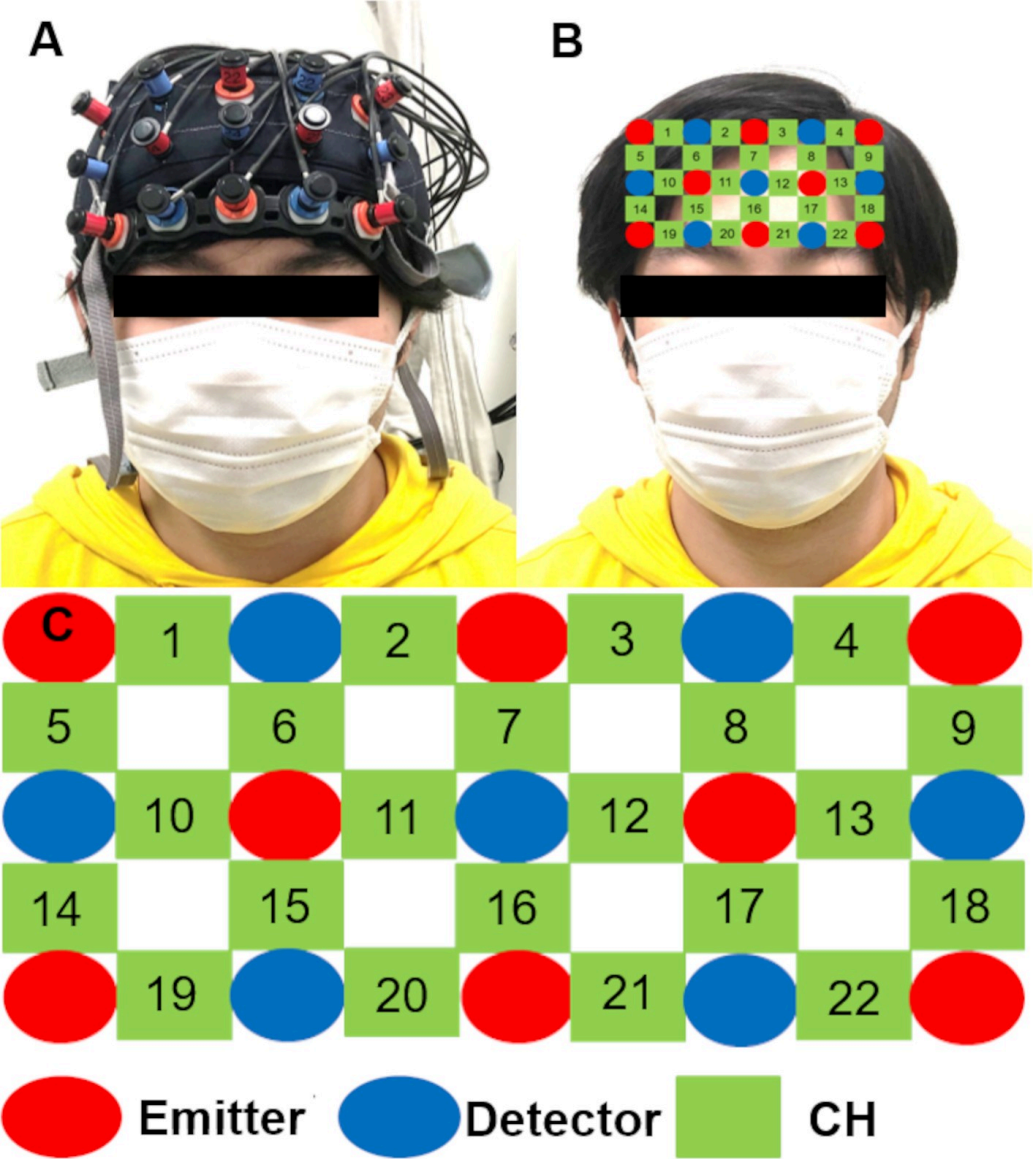
Near-infrared spectroscopy (NIRS). ***A*.** A picture of a typical participant equipped with NIRS optodes. ***B and C***. The NIRS comprised 8 elements for the NIR light emitter (red) and 7 elements for the detector (blue). The numbers indicate the channels (green) recorded. The NIRS channels were placed above the dorsolateral prefrontal cortex and the orbitofrontal cortex. The channels between the optodes measured on the skull were assigned to the brain regions using the virtual registration method.

Before and after patch treatment, NIRS (ETG-4000/OT-R40, Hitachi, Tokyo, Japan) was used to measure the relative concentration of oxyhemoglobin flow over the dorsolateral prefrontal cortex (DLPFC) and orbitofrontal cortex (OFC) (Fig 3). The period between the onset of patch adhesion and the NIRS measurement was about 10 min. The directions of the start and end of posture that produced the pain were given to the participants in the Pain group with an audio guide. The start guidance was given 30 s later after the onset of a NIRS measurement, and the end guidance was given additional 10 s later. For the no-Pain group, although no posture was taken, the same audio guide was played to eliminate the effect of the voice. NIRS channels (CH) 1 ‒ 9 were assigned to the DLPFC, and CH10 ‒ 22 were assigned to the OFC. During the NIRS measurements, the room temperature was maintained at 22–23˚C. The room had white walls and was kept very quiet. The participants wore a sleeping mask to eliminate extra visual stimulation. Details of NIRS measurements were described previously [15]. Briefly, the NIRS optodes were placed in the proper position for each participant according to the international 10–20 system [27]. The channels between the optodes measured on the skull were assigned to brain regions using the virtual registration method [28, 29]. The DLPFC roughly corresponds with Brodmann area 46 [30], and the OFC consists of Brodmann areas 10 and 11 [31]. According to the atlas by Talairach and Tournoux [32], the upper DLPFC was assigned to CH1, 2, 3, and 4; the right DLPFC to CH10, 14; the left DLPFC to CH13, 18; the prefrontal pole to CH11, 12, 15, 16, 17; and the OFC to CH20, 21. The NIRS data were analyzed with MATLAB (2018, MathWorks, Natick, MA, USA). The sampling rate of the NIRS was 10 Hz. All NIRS data were processed using high band-pass (0.001 Hz) and low band-pass (1 Hz) filters to remove artifacts, such as heartbeat and body movements. The NIRS data were averaged using a moving average, and the values of the 3 trials were averaged. The NIRS data were normalized with Z scores for the 30-s periods between the measurement onset and the timing at which the participants sensed pain while assuming the specified pain-producing posture. This normalization allowed us to compare the changes in oxyhemoglobin levels in the specific brain regions while pain was being sensed, and minimized the contribution from the hemodynamic activity in the scalp to the NIRS data measured because its contribution is almost constant in spite of external stimuli [33]. Thus, the NIRS data were considered to represent the oxyhemoglobin levels in our results. The experiments were performed in the same manner for both groups, except the no-Pain group did not assume a posture that evoked pain.

#### ELISA for salivary oxytocin

Before and after patch treatment, saliva was collected from all the participants in both groups. To evoke saliva secretion, the participants chewed a saliva-test gum (Oral Care, Tokyo, Japan) for 5 min. During the first 1 min, the unstimulated saliva was swallowed. The saliva secretion for the next 4 min and gum were then collected in a 50-mL tube. The tube was iced, and the saliva was dispensed in 1-mL aliquots into 1.5-mL tubes and maintained at -20°C. Before oxytocin measurements, the saliva was centrifuged at 1500 ×*g* for 15 min at 4°C, and the supernatant was diluted 10 times with the assay solution of the oxytocin ELISA kit (ADI-901-153A, Enzo Life Sciences, Farmingdale, NY, USA). The salivary oxytocin levels were measured according to the manufacturer’s instructions. This assay was a competitive ELISA [34]. After overnight incubation at 4°C, the excess reagents were washed off and the bound oxytocin phosphatase was incubated with the substrate. After 1 h, the enzyme reaction was stopped, and the optical density was read on a microplate reader Corona Electric SH-1000 (Hitachinaka, Ibaraki, Japan) at 405 nm. The optical density was inversely proportional to the concentration of salivary oxytocin. The content (pg/mL) was determined by plotting the optical density of each sample against a standard curve. The measurement range was 15‒1000 pg/mL according to the manufacturer.

### Statistical analysis

The data are expressed as mean ± SEM or with box-and-whisker plots. Significant differences were defined as *P* < 0.05. Wilcoxon signed-rank test was used as a non-parametric test to compare the paired two groups. Data analysis was performed using R software version 4.10 (https://www.r-project.org/).

## Results

### Quantitative measurements of pain relief by TCES and VAS

**Fig 4:**
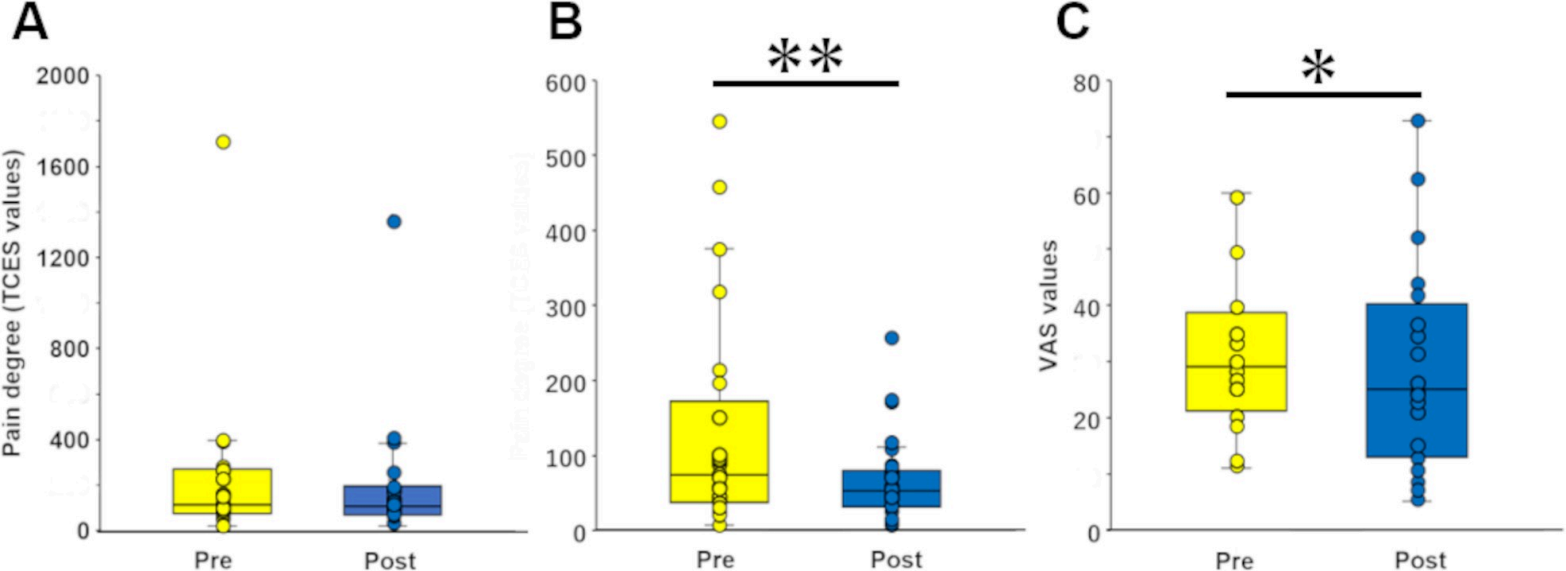
Changes in TCES and VAS values due to patch treatment. ***A*.** TCES values did not change between before (Pre) and after (Post) patch treatment for the participants without pain (no-Pain group). ***B*.** Pain degree obtained by TCES decreased after patch treatment for the participants with pain (Pain group). Wilcoxon signed-rank test, **P < 0.01. ***C*.** The VAS values decreased after patch treatment in the participants with pain (Pain group). Wilcoxon signed-rank test, *P < 0.05.

For the no-Pain group, the differences between the minimum TCES current to evoke pain and the minimum TCES current to be perceived were measured before and after the patch treatment. The TCES values (*i*.*e*., ‘pain degree’ described in the Methods section) for the no-Pain group were 243.1 ± 91.7 and 202.1 ± 71.2 in these 2 trials, respectively (n = 18, Wilcoxon signed-rank test, *P* = 0.325, *Z* = 1.0, Cliff’s delta = 0.01; Fig 4A). These 2 trials corresponded to those before and after the patch treatment for the Pain group. The pain degree values, however, depended largely on the individuals, and thus the data were normalized by the first trial. The normalized values for the second trial were 96.0 ± 0.1% (*i*.*e*., those for the first trial were 100%). That is, there were no significant differences between the 2 trials (n = 18, Wilcoxon signed-rank test, *P* = 0.442). The VAS measurements were not applied to the no-Pain group because the participants were not experiencing pain.

For the Pain group, the TCES values (*i*.*e*., pain degree) were measured when the participants assumed the pain-evoking posture. These values decreased from 130.6 ± 28.9 to 64.5 ± 11.0 following pyramidal thorn patch treatment (n = 27, Wilcoxon signed-rank test, *P* = 0.005, *Z* = 2.8, Cliff’s delta = 0.36; Fig 4B). The normalized values for the second trial were 73.7 ± 0.5% (n = 27, Wilcoxon signed rank test, *P* = 0.006). The VAS values were 31.4 ± 2.9 before the pyramidal thorn patch treatment and 27.0 ± 3.4 after the treatment (n = 26). That is, the pyramidal thorn patch treatment significantly reduced the VAS values in the Pain group (n = 26, Wilcoxon signed-rank test, *P* = 0.027, *Z* = 2.2, Cliff’s delta = 0.21; Fig 4C). Thus, the TCES as well as the VAS showed a significant reduction of pain following pyramidal thorn patch treatment.

### Change in oxyhemoglobin measured by NIRS

**Fig 5:**
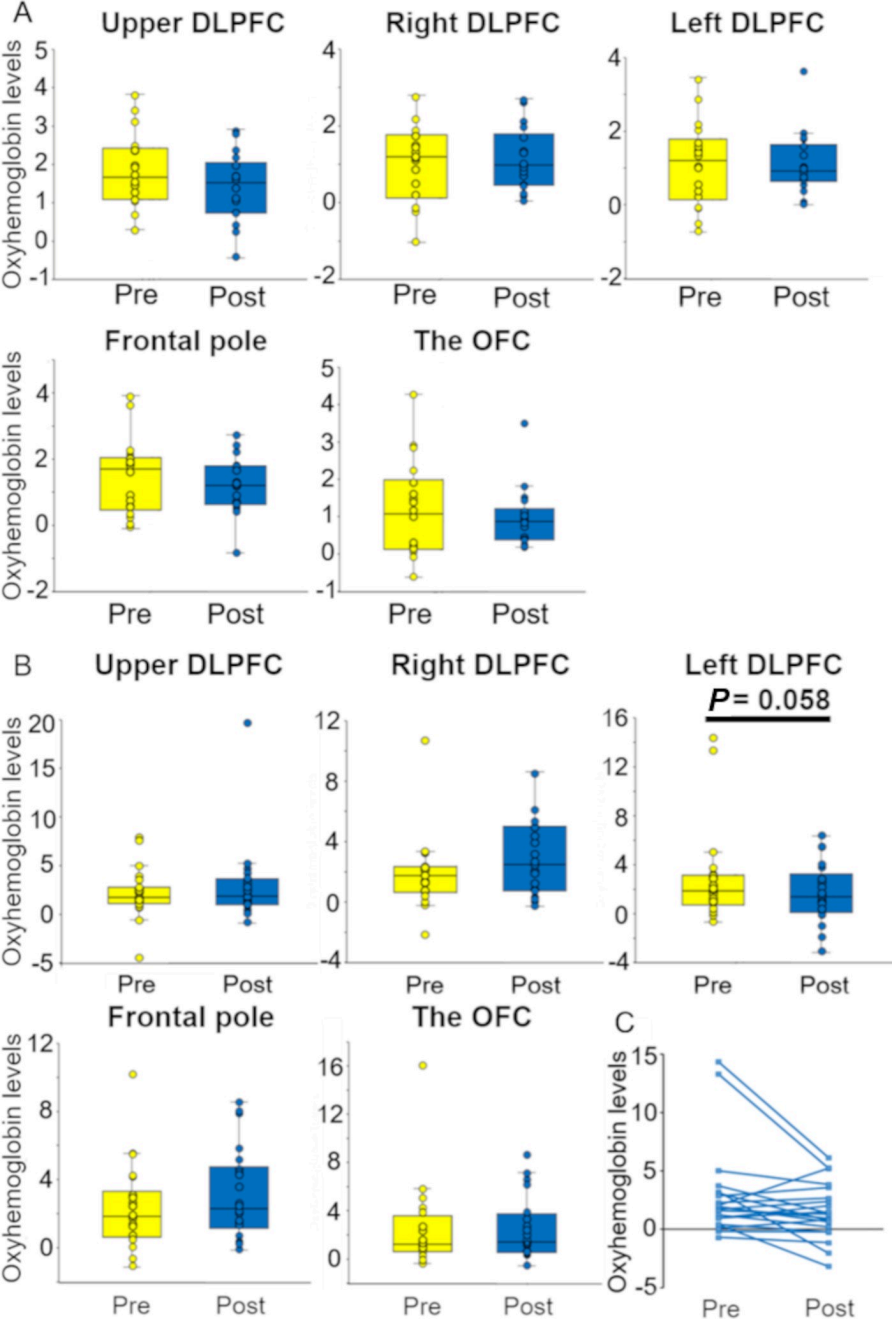
Changes in oxyhemoglobin levels normalized with Z-score before (Pre) and after (Post) pain treatment. ***A*.** Participants without pain (no-Pain group, n = 18). No significant difference was found by Wilcoxon signed-rank test (*P* = 0.325). The DLPFC comprised the upper region, right region, left region, and frontal pole. The directions of posture were not given to the participants in no-Pain group. That is, only the effects of patch adhesion were examined before (Pre) and after (Post) patch treatment. ***B*.** Participants with pain (Pain group, n = 27). In the left DLPFC, there was a tendency of the reduction of oxyhemoglobin levels during patch treatment (n = 21, Wilcoxon signed-rank test, *P* = 0.058). This reduction was supported by the correlation coefficient (*r* = 0.68) calculated for the left DLPFC activity between before patch treatment (*i*.*e*., Pre) and after patch treatment (*i*.*e*., Post). The directions of the start and end of posture that produced the pain were given to the participants in Pain group with an audio guide. ***C*.** All the data (n = 21) recorded in the left DLPFC of the participants with pain (Pain group) before (Pre) and after (Post) pain treatment.

The NIRS experiments were performed in the same way for both groups, except that participants in the no-Pain group did not assume a pain-evoking posture. The oxyhemoglobin levels in the no-Pain group measured by NIRS were not significantly altered in the prefrontal cortex before and after the patch treatment (n = 18, Wilcoxon signed-rank test for each brain region; Fig 5A).

In the Pain group, the oxyhemoglobin level in the left DLPFC (CH13 and 18) had a tendency to decrease after the patch treatment (n = 21, Wilcoxon signed-rank test, *P* = 0.058, *Z* = 1.9, Cliff’s delta = 0.10). Furthermore, the correlation coefficient for the left DLPFC activity between before the patch treatment (*i*.*e*., pre) and after the patch treatment (*i*.*e*., post) showed a correlation (*r* = 0.68). Although NIRS data were obtained from all the participants in the Pain group (n = 27), but the NIRS data of the left DLPFC in 6 patients failed to record. The oxyhemoglobin levels in the other DLPFC regions (such as upper DLPFC (CH1, 2, 3, and 4: *P* = 0.412), right DLPFC (CH10 and 14; *P* = 0.212), frontal pole (CH11, 12, 15, 16, and 17; *P* = 0.390), and the OFC (CH20 and 21; *P* = 0.823) were not significantly altered, although there was a tendency to increase after treatment (Fig 5B and 5C). Thus, we conclude that the left DLPFC was likely to be inactivated by pain relief.

### Salivary oxytocin measurements

We expected that the amount of oxytocin secreted from the posterior pituitary would increase during pain relief in participants sensing pain. To examine this, we performed 2 sets of experiments in both the no-Pain and Pain groups. For the no-Pain group, the pyramidal thorn patches (~15 patches) were applied to the shoulder for 30 min. Before and after the patch adhesion, with an interval of approximately 50 min including the 30-min period of patch adhesion, we obtained the saliva and measured the oxytocin levels by ELISA. The basal oxytocin levels were very low (*i*.*e*., before patch adhesion), and only 2 of the 13 participants without pain had oxytocin levels above the detection limit (15.0 pg/mL). The change in the oxytocin levels following patch adhesion ranged from 42% to 290%. As described above, however, the basal levels were very low, and thus the changes due to patch adhesion were not analyzed further.

**Fig 6:**
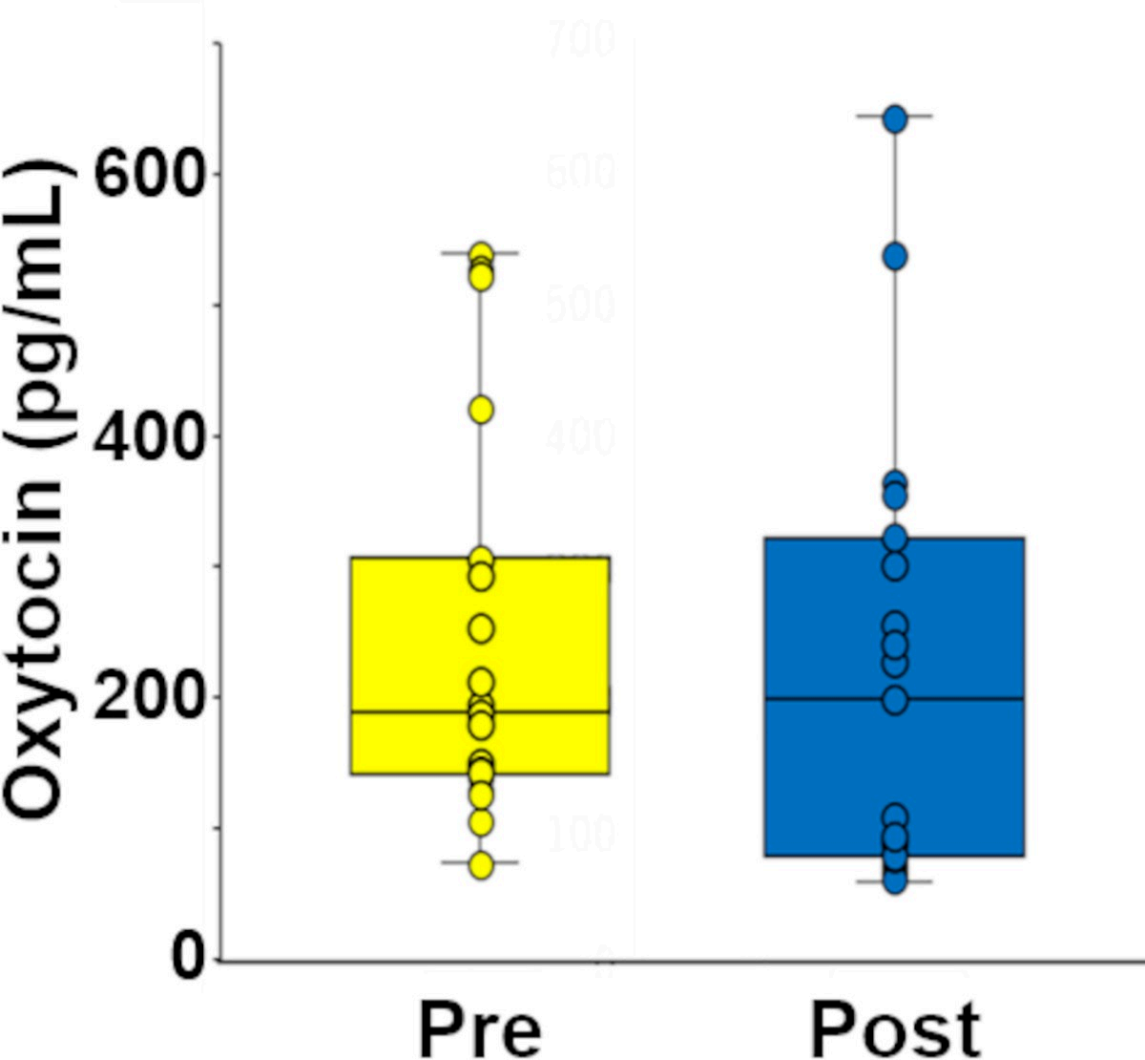
Change in the salivary oxytocin level in saliva during pain relief. Among the 27 participants in the Pain group, the data obtained from 19 patients were within the linear detection range of 15‒1000 pg/mL according to the manufacturer. No significant difference was detected by Wilcoxon signed-rank test (*P* = 0.258).

We also examined the salivary oxytocin levels in the Pain group before and after patch treatment. The time difference between the 2 samplings was the same as for the no-Pain group (50-min interval including the 30-min period of patch adhesion). Among the 27 participants in the Pain group, the saliva obtained from 19 patients showed oxytocin levels within the detection range (15‒1000 pg/mL). We found no significant difference in the salivary oxytocin levels before and after the patch treatment (Wilcoxon signed-rank test, *P* = 0.258, *Z* = 1.17, Cliff’s delta = 0.17; Fig 6).

## Discussion

In the present study, the NIRS data suggested that the left DLPFC was inactivated by pain relief resulting from the thorn patch treatment. This finding is consistent with previous findings that compression at myofascial trigger points, known as ischemic compression, relieves musculoskeletal pain at the neck and that compression decreases the activity in the prefrontal cortex as observed by NIRS [35]. The effects of compression at myofascial trigger points are thought to be mediated by inhibitory effects on prefrontal cortex activity, *i*.*e*., inhibition of hyperactivity of the sympathetic nervous system [35]. On the other hand, suppression of the left DLPFC activity by pain relief in our study appeared to be inconsistent with findings from other studies showing that noninvasive stimulation (*e*.*g*., repetitive transcranial magnetic stimulation) of the left DLPFC effectively treats chronic pain [36–39]. Repetitive transcranial magnetic stimuli applied to the left DLPFC increases oxyhemoglobin levels in the left DLPFC [40].

Our potential explanation for the large difference between our experiments and previous experiments using repetitive transcranial magnetic stimulation is the timing of sensing pain relief in the procedures. In our experiments, we applied the patches to the pain regions and then recorded the oxyhemoglobin levels in the left DLPFC. In the previous experiments, however, the repetitive transcranial magnetic stimuli were applied to the left DLPFC, and then pain relief experienced by the participants was evaluated some weeks after the onset of treatment. In addition, the number of stimuli largely differed between our protocol of patch treatment and the protocol of repetitive transcranial magnetic stimulation.

Furthermore, Morikawa et al. reported that the compression at ‘non’-myofascial trigger points increased the oxyhemoglobin concentration in the prefrontal cortex [35]. That is, the effects of compression at myofascial trigger points closely resemble those of our patch treatment, whereas the effects of compression at non-myofascial trigger points may resemble those of repetitive transcranial magnetic stimulation on the prefrontal cortex. The oxyhemoglobin levels after pain treatment on the basis of repetitive transcranial magnetic stimulation have not yet been fully measured. Here, we propose that repetitive transcranial magnetic stimulation includes some effects of inactivation of the left DLPFC that have not been appropriately assessed. In the near future, we will use both transcranial magnetic stimulation and NIRS measurements to assess the left DLPFC of participants with pain, and carefully observe the changes in oxyhemoglobin levels after stimulation.

Other previous studies showed that chronic pain (*e*.*g*., chronic back pain, migraine, trigeminal neuropathic pain, hypnic headache, chronic post-traumatic headache, hip osteoarthritis, and complex regional pain syndrome) is associated with a decrease in the gray matter of the left DLPFC (probably due to neuronal loss in the left DLPFC) and that successful interventions could reverse this structural abnormality [39, 41]. In addition, on the basis of fMRI studies revealing that painful touch produces greater activation of the OFC than affectively neutral stimuli [42–44], the involvement of the OFC in pain relief should also be considered.

As can be seen in the Results section and S1 File, the *P* values by Wilcoxon signed-rank test for the PFC regions in Fig 5 were: *P* = 0.412 for the upper DLPFC, *P* = 0.212 for the right DLPFC, *P* = 0.058 for the left DLPFC, *P* = 0.390 for the frontal pole, and *P* = 0.823 for the OFC. Only the data of left DLPFC showed the tendency of a change (*i*.*e*., *P* was close to 0.05), but the data obtained from the other regions did not show such a tendency because the *P* values were over 0.2. However, in general, the involvement of DLPFC in pain is well known [39]. Seminowicz and Moayedi described that “the structure and function of the DLPFC is abnormal in some chronic pain conditions. Upon successful resolution of pain, these abnormalities are reversed” [39]. Thus, the attention to the DLPFC for pain relief is appropriate, but the further studies are needed.

As far as we know, a previous paper described that the right DLPFC was involved in the modulation of heat pain [45]. In this study, the transcranial direct current stimulation to the right DLPFC was used to examine the conditioned placebo/nocebo cue response to heat pain. On the other hand, there are many studies indicating that the left DLPFC was associated with pain processing [46–51]. At present, we do not know the detailed mechanism why the left DLPFC activity is decreased during pain relief. The structural and functional differences between the left and right DLPFC should be carefully examined in the future.

The pain degree (Fig 4A, 4B) differed between participants with or without pain. The pain degree recorded in the Pain group was obviously lower than that in the no-Pain group. We concluded that the threshold of pain sensing in the Pain group was lower than that of the minimum perceived pain in the no-Pain group. Thus, the participants with pain are considered to be more sensitive to pain that is bearable for those without pain [52, 53].

No significant difference in the salivary oxytocin levels was detected between before and after the pain relief treatment due to the varying levels of oxytocin (Fig 6). Not our team but the other research team has succeeded in detecting the change in salivary oxytocin levels during meditation using an ELISA kit [34]. In their experiments, the oxytocin levels were measured in participant saliva before and after the meditation, and the salivary oxytocin levels were significantly increased after the meditation. Thus, the validity of the use of an oxytocin ELISA kit has been confirmed. Previous studies clearly showed that oxytocin has antinociceptive and analgesic effects [17, 18]. To achieve these effects, increased oxytocin is thought to be secreted from the posterior pituitary [54, 55]. We consider that some of the secreted oxytocin is consumed for pain relief; thus, the salivary oxytocin levels would vary widely. In addition, the metabolism of oxytocin is rapid [56]. Previous studies showed that the half-life of oxytocin in the blood and cerebrospinal fluid is 5 min and 20 min, respectively [57, 58]. Therefore, because the saliva was collected over 20 min after adhesion of the pyramidal thorn patches, it is possible that the salivary oxytocin was already metabolized at the time of the measurement.

The present results demonstrated that adhering pyramidal thorn patches on the pain region relieved pain. Here, we should consider sham experiments for patch treatment. The sham patch is a patch without a pyramidal thorn. Previous reports demonstrated that analgesic effects were achieved with sham patches, probably due to hair deflection on hairy skin [3]. Thus, the effects of sham patches were included in the pain relief by patch treatment. Furthermore, we should discuss the point that the left DLPFC might be ‘activated before the patch treatment’, because the participants could ‘expect the pain relief by the patch treatment’ [59, 60]. We cannot deny this possibility at present. This point will be examined in the next studies.

In conclusion, the degree of pain relief brought about by patch treatment was qualitatively expressed as a change in the TCES values. The region in the prefrontal cortex involved in this pain relief was the left DLPFC, which was likely to be inactivated by pain relief. The change in salivary oxytocin levels during pain relief varied, probably due to the balance between the secretion of oxytocin from the brain and the utilization of oxytocin at the pain regions. The findings of the present study suggest that the left DLPFC is a target for analgesia in the brain.

## Supporting information

S1 File This is the raw data for Figs 4–6. (XLSX)
